# BLACK STRUCTURAL RESILIENCE ACROSS THE LIFESPAN

**DOI:** 10.1093/geroni/igae098.3026

**Published:** 2024-12-31

**Authors:** Boeun Kim, Alicia Cooke, Tiffany Riser, Sarah Szanton

**Affiliations:** University of Iowa, Iowa, United States; Johns Hopkins University, Baltimore, Maryland, United States; Johns Hopkins University, Baltimore, Maryland, United States; Johns Hopkins University, Baltimore, Maryland, United States

## Abstract

Resilience, which is the ability to resist, recover, or adapt in response to adverse events, can be cultivated through interactions with societal, community, family, and individual factors across the life course. However, structural resilience, signifying the extent of recruiting resources at societal and community levels, has been overlooked. Furthermore, no study has examined structural resilience using a life course approach, although structural resilience is likely to change over time. This study employed a qualitative descriptive design to understand better the structural resilience and their supportive roles in maintaining or promoting health over the life course through older African Americans’ lived experiences. This study focused on African Americans, who were known to have higher levels of individual resilience than White Americans, to learn what resources helped them to thrive despite adverse circumstances. We conducted semi-structured interviews with 15 self-identified African American adults aged 50 and older living in Baltimore City. Two study team members coded the transcripts using an inductive approach, and themes were generated from the constant comparative approach. The five themes emerged: 1) underwent extraordinary adversities over the life course, 2) accessible resources during childhood, adulthood, and late adulthood, 3) emergence of grassroots organizations to provide support, 4) roles of structural resilience in response to adversities, and 5) dynamics of structural resilience across the life course. This study contributes to a better understanding of the mechanisms that preserve health under stressors. The findings may help to resolve the public health tragedy of health equity faced by older African Americans.

